# The Unfinished Ecosystem: Why Remote Patient Monitoring Has Matured Unevenly, and What Closing the Gap Will Require

**DOI:** 10.3390/healthcare14121698

**Published:** 2026-06-14

**Authors:** Temitope S. Ajagbe

**Affiliations:** Safeguard Medix, San Antonio, TX 78258, USA; temi@safeguardmedix.com; Tel.: +1-309-660-1015

**Keywords:** remote patient monitoring, digital health, telemedicine, reimbursement, professional liability, data privacy, health equity, patient engagement, adherence, chronic-disease management, artificial intelligence in healthcare, federated learning, virtual ward, COVID-19

## Abstract

**Highlights:**

**What are the main findings?**

- Remote patient monitoring (RPM) has matured unevenly: specific applications now generate measurable clinical and economic value, but four cross-cutting structural gaps—provider economics, professional liability, patient privacy and equity, and engagement and adherence—prevent that value from generalising across the field.
- The COVID-19 emergency briefly suspended much of the friction in this ecosystem and produced a useful natural experiment: programmes that scaled rapidly under emergency conditions and then atrophied as those conditions ended demonstrate that the field’s binding constraints are economic and institutional, not technological.

**What are the implications of the main findings?**

- Engagement and adherence should be reframed as a structural pillar of RPM ecosystem maturity—a primary outcome and a reimbursable activity—rather than as a residual or a secondary endpoint.
- A six-point research and policy agenda—economic-evaluation standardisation, work-based reimbursement, profession-wide liability and competency standards (including for AI- and federated-learning-mediated RPM), privacy and benefit as design requirements, engagement as a primary outcome, and deliberate investment in underserved settings—is required to move RPM from localised successes to a trustworthy, generalisable standard of care.

**Abstract:**

Remote patient monitoring (RPM) is widely framed as a foundational technology for the next generation of chronic-disease care. Specific applications—pacemaker follow-up, hypertension cohorts, structured heart-failure programmes, post-surgical biosensor protocols, and virtual wards—now generate measurable clinical and economic value. Yet a decade of evaluations and implementation studies suggests that the surrounding ecosystem has matured unevenly: working applications coexist with persistent cross-cutting fragility. In this Perspective we argue that four structural gaps continue to constrain RPM’s promise at scale: (i) economic models that do not credibly compensate the asynchronous clinical work that RPM generates; (ii) ambiguous frameworks for professional liability and accountability for continuous data streams, intensified by artificial-intelligence (AI)-mediated decision support; (iii) privacy, equity, and benefit-sharing arrangements that do not yet make patients unambiguous net beneficiaries—a gap visible across very different health systems internationally; and (iv) engagement and adherence dynamics that determine whether programmes deliver value at all, but are still treated as secondary outcomes. The COVID-19 emergency briefly suspended much of the friction in this ecosystem and produced a useful natural experiment: what scaled rapidly under emergency conditions, and what subsequently atrophied, illuminates which gaps are technical, which are economic, and which are institutional. We close with a six-point research and policy agenda intended to move RPM from localised successes to a trustworthy, generalisable standard of care.

## 1. Introduction: An Uneven Maturation

Remote patient monitoring (RPM) has accumulated, over more than a decade, a body of evidence that is at once encouraging and curiously inconclusive. Systematic and scoping reviews report improvements in selected clinical endpoints, plausible reductions in healthcare utilisation for some chronic conditions, and growing patient acceptance across an expanding range of indications [1,2,3]. Specific applications—among the strongest examples in the current evidence base—now generate measurable value: large retrospective cohorts demonstrate improved outcomes in hypertension [4]; pacemaker remote follow-up shows favourable cost profiles relative to in-office visits [5,6]; structured heart-failure programmes and inpatient virtual wards report operational gains [7,8]; and biosensor-supported post-surgical monitoring is feasible at the bedside [9]. RPM is no longer experimental in these settings.

And yet, every recent synthesis qualifies its enthusiasm with the same recurring caveats: heterogeneous economic evaluation, fragile reimbursement, ill-defined clinical workflows, unresolved liability questions, uneven equity, and unsustained patient engagement [10,11,12,13]. The pattern is striking. Where RPM works, it works concretely; where the field as a whole is judged, it remains structurally incomplete.

This perspective argues that the apparent paradox is not a paradox at all. RPM has matured technologically faster than its surrounding ecosystem has matured economically, legally, and ethically—and that maturation has been uneven across applications and across health systems. The technical layer is broadly capable, though its maturity varies considerably across device categories, clinical conditions, AI-enabled platforms, connectivity contexts, and health-system settings: devices, connectivity, and AI-mediated analytics are sophisticated and improving rapidly in well-resourced environments, with more uneven progress elsewhere [14,15]. What has not generalised is the surrounding scaffolding. Four structural gaps continue to constrain the field’s impact at scale: (i) provider economics; (ii) professional liability and accountability; (iii) patient privacy, equity, and net benefit; and (iv) engagement and adherence. These gaps interact: poor economics depresses the staffing required for engagement, ambiguous liability discourages the asynchronous workflows engagement requires, and privacy–equity failures shape who gets enrolled in the first place.

I develop this argument in four substantive sections, drawing on a representative body of recent literature spanning rural and urban deployments, perioperative pathways, neuropsychiatric and paediatric care, AI-augmented platforms, and a notable diversity of health systems internationally—Italy, Romania, Portugal, Cameroon, Saudi Arabia, Sudan, Israel, Australia, the United States, and elsewhere. I make particular use of the COVID-19 era as a natural experiment in which much of the normal economic and regulatory friction in RPM was briefly suspended, allowing the ecosystem’s real load-bearing structures to become visible. Section 2 examines the provider-economics gap. Section 3 addresses professional liability, including the new accountability questions that AI-mediated decision support introduces. Section 4 considers privacy, equity, and net benefit in international perspective. Section 5 introduces engagement and adherence as a distinct fourth pillar. Section 6 proposes a six-point research and policy agenda, and Section 7 concludes.

### Methodological Note on the Literature Synthesis

This Perspective draws on a narrative synthesis rather than a systematic review. Sources were identified through three complementary strategies: (i) targeted searches of PubMed, Scopus, and the MDPI Healthcare archive using combinations of the terms “remote patient monitoring”, “telemonitoring”, “digital health”, “implementation”, “economic evaluation”, “engagement”, “adherence”, “liability”, “AI”, “federated learning”, “equity”, and “COVID-19”, restricted to 2015–2026 publications and supplemented by earlier seminal work where relevant; (ii) snowball sampling from the reference lists of recent scoping and systematic reviews [1,2,3,10,11]; and (iii) purposive selection to ensure coverage across the four structural pillars and across diverse clinical domains and international health-system contexts. I included peer-reviewed publications reporting empirical findings, scoping or systematic reviews, qualitative implementation studies, and methodological commentaries; I excluded purely technical engineering papers without an implementation or evaluation dimension. The synthesis is representative rather than exhaustive, and the cited literature is offered as the strongest evidence available for each structural claim rather than as a comprehensive enumeration.

## 2. The Provider-Economics Gap

Most clinical innovations succeed when they make existing work easier or generate revenue commensurate with the new work they require. RPM does neither cleanly. It generates a continuous, asynchronous stream of data that must be reviewed, triaged, documented, and acted upon—work that does not map neatly onto the encounter-based logic of most reimbursement systems. Scoping reviews of perioperative and inpatient RPM consistently describe new monitoring tasks layered onto already-saturated clinical workflows, often without dedicated staffing or compensation models [16,17]. Capacity-planning analyses of virtual wards confirm that the staffing implications of continuous monitoring are non-trivial and easily underestimated at programme design [8]. Alternative organisational models—for example, hospital–provider-company partnerships for home non-invasive ventilation—show that the workforce and reimbursement problem is sometimes solved by reallocating responsibilities across organisational boundaries rather than within them [18], a pattern likely to recur as RPM matures.

The economic-evaluation literature reflects this incoherence. A recent scoping review of economic-evaluation methodologies for RPM in chronic conditions found wide methodological heterogeneity, inconsistent analytical perspectives—payer (costs to insurers and payment systems), provider (costs to clinicians and health-system delivery), and societal (the full range of costs, including those borne by patients, families, and the wider economy)—and few studies that adequately capture the indirect costs of clinician time, alert management, and programme administration [10]. Implementation studies make the consequences concrete: academic–community partnerships dedicated to disseminating RPM in primary care report that uptake depends heavily on grant funding, philanthropic support, or temporary policy windows rather than on a self-sustaining reimbursement logic [19], and mixed-methods evaluations of diabetes RPM identify reimbursement uncertainty, staff workload, and unclear billing pathways among the most consistent barriers reported by clinicians [20,21].

Specific cost studies, where they exist, are encouraging but narrow. A Portuguese district-hospital comparison of in-office versus remote pacemaker follow-up demonstrated material cost advantages for remote monitoring once reimbursed transportation was factored in [5]. A long-term Spanish–Norwegian study of pacemaker monitoring quantified previously hidden socioeconomic costs absorbed by informal caregivers—costs that are systematically excluded from conventional health-system perspectives [6]. These studies are valuable precisely because they are unusual; they are not representative of the typical RPM evaluation, which lacks comparable rigour.

At the population level, socioeconomic determinants further skew adoption. Hospitals in rural and lower-resource settings—those whose patients arguably stand to benefit most—are systematically less likely to implement RPM, in part because the underlying economic case is fragile [22]. Earlier rural heart-failure work showed real utilisation benefits but depended on dedicated programme infrastructure that did not generalise [23].

The COVID-19 period is informative here. Multiple Italian and Mediterranean health-system reports document rapid, large-scale RPM and televisit deployment during lockdown—for cardiovascular disease [24,25], cancer follow-up [26], total joint arthroplasty [27], and regional telemonitoring at scale [28]. When normal reimbursement and regulatory friction were relaxed under emergency authority, programmes scaled within weeks. What happened next is the more revealing question: reports suggest that many of these programmes contracted as emergency measures expired, though systematic post-pandemic follow-up evidence remains limited. Where contraction is documented, the bottleneck appears to have been structural rather than technical or clinical: the surrounding economic logic returned. This sequence supports, plausibly though not conclusively, the broader argument that RPM’s constraint is ecosystem-level rather than technology-level.

Until reimbursement explicitly recognises the labour of asynchronous monitoring, decision-support review, and care coordination—and does so in a manner that survives outside emergency declarations—RPM will continue to be financially marginal for the very providers whose participation is required to make it succeed. The current Medicare RPM and remote therapeutic monitoring code families are a useful start, but they remain narrow, billing-condition-specific, and poorly suited to multi-condition or population-level programmes. A mature ecosystem requires reimbursement that follows the data—and the work that the data create—rather than the device.

## 3. The Liability and Accountability Gap

RPM transforms episodic clinical observation into a continuous data stream. This shift carries an under-acknowledged corollary: every datum collected is a datum that, in principle, could have been acted upon. The legal and professional implications of this transformation have not been resolved.

Competency frameworks for sensor- and wearable-based monitoring remain in early development. A scoping review of clinician competencies for RPM identified substantial gaps in training across data interpretation, alarm management, and the boundaries of clinical responsibility for asynchronous data [29]. In specialty domains where the stakes of missed signals are particularly high—perioperative recovery, neonatal and paediatric care, and neuropsychiatric monitoring—reviews repeatedly highlight unresolved questions about how RPM data should be integrated into clinical decision-making and who bears responsibility when it is not [16,30,31].

AI- and IoT-mediated RPM intensifies these questions. The technical literature describes a coherent and rapidly maturing wave: machine-learning architectures for diabetes monitoring [32,33], IoT-based healthcare monitoring systems [34], intelligent IoT devices for infection screening [35], federated-learning frameworks for privacy-preserving prediction [36], and AI applications across mental-health screening, support, monitoring, and prevention [37]. Generative AI is now being explicitly examined in health informatics [38,39]. In heart failure, predictive pathophysiology-driven monitoring is becoming an explicit programme aim rather than an aspiration [40]. These models triage incoming streams, surface anomalies, and recommend escalation, introducing a new layer of agency between patient and clinician—with corresponding ambiguities about explainability, oversight, and shared accountability [14].

Federated learning illustrates the complexity sharply. By keeping training data local while sharing only model updates, it offers a partial answer to the data-residency dimension of the privacy concern [36]. Yet the same architecture introduces new questions: how is model behaviour audited when no central party sees the training data? How is accountability distributed when an algorithmic recommendation, derived in part from a federation of institutions, contributes to a clinical error at one of them? The technical maturation of AI in RPM is not eliminating liability questions; it is multiplying them.

Three concrete illustrations make the accountability problem more tractable. First, when an AI model performs alert prioritisation—escalating some incoming signals, suppressing others—responsibility for a missed event is distributed across the model developer (who set the thresholds and training data), the institution (which adopted and configured the model), and the clinician (who may have relied on the prioritisation in good faith). Second, automated risk-scoring systems that flag deteriorating patients introduce a new form of clinical evidence whose audit trail and confidence intervals may not be fully transparent to the responsible clinician, raising the question of how much weight is reasonable to place on a score the clinician cannot fully interrogate. Third, clinician-in-the-loop escalation models, in which the algorithm recommends and the clinician decides, do not automatically resolve liability: a clinician’s override of a confident AI recommendation, or failure to override an incorrect one, both raise questions that current medico-legal frameworks were not designed to adjudicate. Each of these patterns describes work that is already being done in RPM programmes today, and each is a domain in which the assignment of accountability remains an open institutional question.

Workforce-side perspectives reflect this discomfort. Qualitative interviews with rural and rural-serving primary care providers and cardiologists describe enthusiasm for RPM tempered by concerns about the medico-legal exposure of continuously generated data, particularly when staffing is thin and after-hours coverage is uncertain [41]. Telenursing studies in Israel surface parallel concerns from the nursing workforce—quality, role boundaries, and accountability for asynchronous interactions [42]. Capacity-planning work for virtual wards highlights that the staffing required to safely respond to continuous data is consistently underestimated [8]. Surveys of medical students entering practice indicate broadly positive but cautious attitudes toward AI-integrated telemedicine [43], suggesting that competency development is needed not only for current clinicians but also for the workforce now training.

A mature RPM ecosystem requires explicit, profession-wide standards on what constitutes adequate review of asynchronous data, what response times are reasonable for which alert classes, and how liability is shared across the device manufacturer, the algorithm developer (including federated-learning consortia), the monitoring service, and the responsible clinician. Without such standards, individual clinicians and institutions will continue to absorb risk that none of them can fully characterise.

## 4. The Patient Gap: Privacy, Equity, and Net Benefit in International Perspective

RPM is frequently described as patient-centred. Whether it is also patient-advantageous in the full sense—improving outcomes, protecting privacy, and distributing benefits fairly—is a more delicate question, and the answer varies considerably across health systems. These three dimensions interact but remain analytically distinct: privacy concerns the conditions under which monitoring is conducted, equity concerns who is monitored, and net benefit concerns what monitoring delivers. A programme can satisfy any one of these criteria without satisfying the others, and a mature RPM ecosystem must address all three concurrently. The sections that follow consider each in turn.

On privacy, the continuous and intimate nature of RPM data raises concerns that the standard regulatory toolkit was not designed to address. Modern RPM platforms commonly involve a chain of actors—device manufacturers, cloud hosts, algorithm vendors, monitoring services, and providers—each with potential access to data and each operating under partially overlapping rules. Patient-experience studies show that individuals frequently do not understand who sees their data, for how long, and to what end, and that this ambiguity affects their willingness to engage [44]. Co-design research with inpatients similarly highlights that meaningful consent and comprehension cannot be assumed; they must be designed in [45]. User-centred development of digital phenotyping tools in ophthalmology illustrates the level of engagement required to make consent and data flows intelligible to patients [46].

On equity, the literature is unambiguous that RPM is not neutrally distributed, and the international evidence base sharpens the picture. Hospitals serving rural and lower-resource populations in the United States are less likely to implement RPM, even when their patient panels would arguably benefit most [22,41]. A low-cost IoT-based cardiovascular monitoring deployment in Cameroon demonstrates that the technical barriers to RPM in low-resource settings are largely solvable [34]; the limiting factors are infrastructural, regulatory, and economic. Romanian work on remote monitoring for elderly mental and emotional health during and after COVID-19 highlights both the opportunity and the dependency on specific national digital infrastructures [47,48]. A Saudi narrative review of telehealth for chronic obstructive pulmonary disease (COPD) [49] and a Sudanese scoping review of telemedicine in obstetrics and gynaecology [50] reach related conclusions: where structural support exists, RPM extends access; where it does not, RPM risks codifying existing disparities. Specialised populations such as seafarers, addressed by maritime telemedicine, illustrate how RPM can fill genuine access gaps when designed to do so [51]. Connectivity, device cost, and digital literacy compound these structural barriers, particularly for underserved communities [52,53].

On clinical benefit, the picture is encouraging but uneven. Reviews and primary studies consistently identify domains in which RPM appears to improve utilisation or selected outcomes—heart failure [7,40], hypertension [4], diabetes [20,21,32,33], hypertensive disorders of pregnancy [54], asthma in children [55], COPD self-management [49,56], cystic fibrosis care models [57,58], cardiac rehabilitation [59], pacemaker follow-up [5,6], cancer televisits [26], post-arthroplasty management [27], lower-limb amputee rehabilitation [60], and Parkinson’s disease self-assessment [61]—but effect sizes vary, evidence for hard endpoints is thinner than for process measures, and study quality is heterogeneous [1,3,11,23,62]. Health-literacy research adds a further nuance: RPM can either enhance or undermine the therapeutic relationship depending on how it is implemented, with patients valuing it most when monitoring is paired with clear communication and responsive clinical follow-through [44].

Taken together, these strands suggest that the patient-side case for RPM is not yet decisive in the way that its proponents sometimes suggest. Patients can benefit substantially, and many do—but they do so within a framework that does not yet consistently guarantee privacy, equitable access, or unambiguous net advantage across health systems with very different starting positions. A mature RPM ecosystem must do all three.

To clarify how each of these international cases speaks to the four structural gaps, Table 1 maps cited settings to the gap each primarily illuminates and to the mechanism by which it does so.

## 5. The Engagement and Adherence Gap

A recurring weakness across the literature, easily missed because it is rarely the headline finding, is that RPM programmes are unusually sensitive to patient engagement and adherence. To illustrate the structural significance of this sensitivity, a hypothetical monitoring programme with a 40 percent drop-out rate is not 60 percent of a working programme; it is, frequently, an economic loss with attenuated clinical effect and heightened liability exposure for the patients who remain. We argue that engagement deserves to be treated as a structural pillar of RPM ecosystem maturity, not as a secondary outcome.

The empirical signal is consistent. A study of heart-failure remote-monitoring programme drop-out, paired with healthcare-professional perspectives, identified persistent attrition and clinician-reported difficulties in sustaining engagement over time [66]. A mixed-methods evaluation of diabetes RPM enrolment and attrition in a US population reached similar conclusions, with attrition concentrated in subgroups already at higher clinical and social risk [21]. Patient-experience research on RPM in general practice surfaces the underlying mechanism: when monitoring is continuous but clinical response is not, when consent is bureaucratic rather than substantive, or when the technology and the therapeutic relationship are perceived as substitutes rather than complements, engagement attenuates [44]. Co-creation work with COPD patients on smartwatch-based self-management documents the level of inclusive, pragmatic design effort required to sustain meaningful use over time [56]. Smartphone-based care management platforms applied to orthopaedic patients similarly find that engagement is contingent on perceived clinical responsiveness and platform usability [67].

Specialty programmes further illustrate the point. The Co-HIVE depression model demonstrates that virtual assessment and remote monitoring can be received positively by patients when the model integrates monitoring with structured clinical contact rather than substituting for it [65]. Italian work on remote mental-health assessment of pregnant women during the COVID-19 pandemic reaches a comparable conclusion: feasibility is high when the programme is designed for sustained relational engagement rather than data collection alone [63]. Case-management integration in cystic fibrosis units shows that engagement can be substantially strengthened by an explicit human coordination layer between patient and monitoring data [58]. Conversely, programmes that rely on patient self-engagement without such structures consistently report attrition.

Operationalising this commitment requires that engagement be measured with concrete, comparable indicators. We propose six that future RPM studies should report as a minimum: (a) enrolment rate (the proportion of eligible patients who join the programme); (b) active-use rate (the proportion of enrolled patients actively transmitting data at the cadence the programme prescribes); (c) data-transmission completeness (the proportion of expected transmissions actually received); (d) alert-response adherence (the proportion of generated alerts to which the intended response—clinician contact, patient action, or escalation—occurs within target time); (e) attrition by subgroup, with explicit reporting by socioeconomic, demographic, and clinical subgroup so that the field can detect programmes that perform well in aggregate but fail for those most in need; and (f) patient-reported usability and burden. Reporting these indicators consistently would allow the field to distinguish RPM programmes that deliver sustained value across their enrolled populations from those that deliver value only in aggregate while systematically losing the patients least equipped to remain engaged.

Engagement is not a soft variable. It is the operational precondition for every other RPM benefit. A reimbursement model that does not pay for sustained engagement work pays for monitoring that frequently does not occur. A liability framework that does not address the responsibilities of the engagement layer leaves both clinicians and patients exposed to ambiguity at precisely the point where most adverse events would arise. A privacy-and-equity framework that does not consider the literacy, language, and infrastructural conditions for engagement systematically excludes the populations RPM is most often claimed to serve. Treating engagement as a fourth pillar—rather than as the residual of the other three—is the structural correction the field requires.

## 6. Toward a Mature RPM Ecosystem: A Six-Point Agenda

If the four gaps that we have identified are structural rather than incidental, then closing them requires coordinated effort across research, policy, clinical practice, and industry. We suggest six priorities.

First, standardise the economic evaluation of RPM. The methodological heterogeneity documented in recent reviews [10] is solvable. Reference frameworks should specify time horizons, perspectives (including the informal-caregiver and societal perspectives that current methods routinely omit [6]), included costs (especially the labour of asynchronous review), and counterfactual definitions, so that programmes can be compared and reimbursement decisions made on a stable evidentiary base.

Second, tie reimbursement to the work, not the device. Coding structures should compensate for the cognitive labour of clinicians and care teams who review and act on RPM data, including cross-condition and population-level monitoring, rather than tying payment to narrow device-specific pathways. The COVID-19 natural experiment demonstrated that RPM scales rapidly when economic friction is reduced; the policy task is to construct a stable post-emergency reimbursement that retains that responsiveness without depending on emergency authority [24,25,26,27,28].

Third, develop explicit liability and competency standards, including for AI- and federated-learning-mediated RPM. Professional societies, regulators, and indemnifiers should converge on what constitutes adequate review of asynchronous data, what response times apply to which alert classes, and how responsibility is distributed across the manufacturer, algorithm developer (including federated consortia), monitoring service, and clinician—building on but extending existing competency frameworks [29] and addressing the specific accountability questions that predictive and generative AI raise [14,36,38,40].

Fourth, treat patient privacy and benefit as design requirements, not afterthoughts. Co-design methodologies [45,46] and patient-experience research [44] provide a starting point. Privacy notices, data-sharing terms, and benefit-sharing arrangements should be intelligible, auditable, and revisable, and equity should be measured as a primary outcome of RPM implementations across the diverse health systems in which the technology now operates [47,48,49,50,51,64].

Fifth, treat engagement and adherence as a primary structural concern. Engagement should be a reportable outcome in evaluations, a design target in implementations, and a reimbursable activity in payment models. Drop-out should be analysed by subgroups so that the field can detect when programmes work in aggregate but fail for the populations most in need [21,56,66].

Sixth, invest deliberately in implementation in underserved settings. The populations most likely to benefit from RPM are systematically the least likely to receive it [22,41,52]. Closing this gap requires investment in connectivity, device subsidies, multilingual interfaces, and trust-building with communities for whom digital health is not a default expectation—and it requires recognising that low-resource health systems internationally have already demonstrated technical feasibility [34] when the surrounding ecosystem permits it.

Table 2 summarises the relationship between the six agenda items and the four structural gaps. Most agenda items address more than one gap, which is itself part of the argument: structural maturation in RPM cannot be achieved by addressing each gap in isolation, because the gaps interact.

## 7. Conclusions

RPM is not a failed technology; it is an unevenly matured one. This perspective offers a conceptual and policy-oriented synthesis rather than an empirical analysis; its contribution lies in proposing a structural framework for understanding why a technologically mature field continues to deliver uneven outcomes, and in identifying the institutional conditions under which that unevenness might be addressed. The clinical evidence is sufficient to justify continued investment, and in many specific contexts—pacemaker follow-up, hypertension cohorts, structured heart-failure programmes, post-surgical biosensor protocols, virtual wards, cardiac rehabilitation—it is sufficient to justify scaling. But the ecosystem around the technology has not matured at the same pace. The economics that determine whether providers can sustain it, the liability framework that determines whether they can responsibly run it, the privacy and equity arrangements that determine whether patients are genuine beneficiaries, and the engagement structures that determine whether programmes deliver value at all—these have advanced unevenly and remain the binding constraints on the field. Acknowledging this is not a critique of RPM’s promise. It is, we believe, the precondition for realising it.

## Figures and Tables

**Table 1:** International RPM cases mapped to structural gaps.

Country/Setting	Cited As	Primary Gap Illuminated	Mechanism by Which the Case Illuminates the Gap
United States (rural hospitals)	[22,41]	(iii) Equity; (i) Economics	Adoption depressed by fragile economic case; rural cardiologists and primary care providers cite medico-legal exposure as a barrier.
Cameroon (low-cost IoT cardiovascular monitoring)	[34]	(iii) Equity	Demonstrates technical feasibility in a low-resource setting; the binding constraints are infrastructural, regulatory, and economic rather than technological.
Romania (elderly mental and cognitive health)	[47,48]	(iii) Equity (digital infrastructure); (iv) Engagement	Dependency on national digital infrastructure shapes both reach (who is enrolled) and sustained engagement (who remains active).
Saudi Arabia (COPD telehealth)	[49]	(iii) Equity; (iv) Engagement	Access extension and sustained self-management are contingent on structural support; outcomes depend on the surrounding service model.
Sudan (obstetrics and gynaecology telemedicine)	[50]	(iii) Equity	Telemedicine extends access only where structural support exists; otherwise, it risks codifying disparities.
Italy (COVID-era RPM at scale; perinatal mental-health monitoring)	[24,25,26,27,28,63]	(i) Economics; (iv) Engagement	Rapid scaling under emergency authority followed by contraction post-emergency; programmes sustaining engagement did so through relational design, not data collection alone.
Portugal (pacemaker remote follow-up costs)	[5]	(i) Economics	Concrete cost advantage demonstrated when reimbursed transportation is included in the cost frame.
Spain/Norway (informal caregiver costs of pacemaker monitoring)	[6]	(i) Economics	Societal-perspective evaluation reveals informal-caregiver costs systematically omitted by conventional health-system perspectives.
Israel (telenursing perceptions)	[42]	(ii) Liability and accountability	Nursing-workforce concerns about quality, role boundaries, and accountability for asynchronous interactions surface the under-specified liability layer in non-medical clinical roles.
Australia (HIVE virtual ward; Co-HIVE depression model)	[64,65]	(iv) Engagement; (ii) Liability	Operational viability of monitoring combined with structured clinical contact; positive patient experience when monitoring is paired with explicit relational engagement.
Seafarers (maritime telemedicine)	[51]	(iii) Equity	Telemedicine designed for specialised populations fills genuine access gaps that conventional care cannot reach.

Roman numerals (i–iv) refer to the four gaps defined in Section 1: (i) provider economics; (ii) professional liability and accountability; (iii) patient privacy, equity, and net benefit; (iv) engagement and adherence.

**Table 2:** Structural gaps × research and policy agenda.

Research and Policy Agenda Item	(i) Economics	(ii) Liability	(iii) Privacy/Equity	(iv) Engagement
(1) Standardise economic evaluation of RPM	●		○	○
(2) Work-based reimbursement (pay for the work, not the device)	●	○		●
(3) Liability and competency standards (including for AI- and federated-learning-mediated RPM)	○	●	○	○
(4) Privacy and benefit as design requirements		○	●	○
(5) Engagement as a primary outcome and reimbursable activity	○	○	○	●
(6) Investment in implementation in underserved settings	●		●	●

● denotes a primary mechanism by which the agenda item closes the gap; ○ denotes a secondary or supporting mechanism. Gap columns refer to: (i) provider economics; (ii) professional liability and accountability; (iii) patient privacy, equity, and benefit; (iv) engagement and adherence.

## Data Availability

No new data were created or analysed in this study. Data sharing is not applicable to this article.
